# miR-25 modulates triacylglycerol and lipid accumulation in goat mammary epithelial cells by repressing *PGC-1beta*

**DOI:** 10.1186/s40104-018-0262-0

**Published:** 2018-06-18

**Authors:** Liuan Ma, Huiling Qiu, Zhi Chen, Li Li, Yan Zeng, Jun Luo, Deming Gou

**Affiliations:** 1 Shenzhen Key Laboratory of Microbial Genetic Engineering, College of Life Sciences and Oceanography, Shenzhen University, Shenzhen, 518060 Guangdong China; 2 Biomedical Engineering, Health and Environmental Engineering, Shenzhen Technology University, Shenzhen, 518000 Guangdong China; 3 Shaanxi Key Laboratory of Molecular Biology for Agriculture, College of Animal Science and Technology, Northwest A&F University, Yangling, 712100 Shaanxi China; 4 Present Address: Shenzhen Key Laboratory of Microbial Genetic Engineering, College of Life Sciences and Oceanography, Shenzhen University, Shenzhen, 518060 Guangdong China

**Keywords:** Goat mammary epithelial cells, Lipid, miR-25, *PGC-1beta*, Triacylglycerol

## Abstract

**Background:**

The goat (*Caprahircus*) is one of the most important livestock animals. Goat milk fat is an important component in the nutritional quality of goat milk. Growing evidence points to the critical roles of microRNAs (miRNAs) in lipid metabolism.

**Results:**

Using a highly sensitive method of S-poly(T) plus for miRNAs detection, we analyze the expression patterns of 715 miRNAs in goat mammary gland tissues at different stages of lactation. We observed that miR-25 expression had an inverse relationship with milk production. Overexpression of miR-25 significantly repressed triacylglycerol synthesis and lipid droplet accumulation. To explore the regulatory mechanism of miR-25 in milk lipid metabolism, we analyzed its putative target genes with bioinformatics analysis followed by 3′-UTR assays. Peroxisome proliferative activated receptor gamma coactivator 1 beta (*PGC-1beta*), a key regulator of lipogenics was identified as a direct target of miR-25 with three specific sites within its 3′-UTR. In addition, miR-25 mimics in goat mammary epithelial cells reduced the expressions of genes involved in lipid metabolism.

**Conclusions:**

Taken together, our results show miR-25 is potentially involved in lipid metabolism and we reveal the function of the miR-25/*PGC-1beta* regulatory axis during lactation.

**Electronic supplementary material:**

The online version of this article (10.1186/s40104-018-0262-0) contains supplementary material, which is available to authorized users.

## Background

The goat (*Caprahircus*) is an important provider of meat and dairy products. Goat milk contains larger amounts of capric, caprylic and medium-chain fatty-acids and smaller globules [1]. These increase the digestibility of goat milk and may promote positive health effects [2]. Analysis of the human consumption of goat and cow milk fat showed that goat milk reduced cholesterol levels but not levels of triglycerides, high-density lipoprotein cholesterol, glutamic oxaloacetic transaminase or glutamic pyruvic transaminase [3]. Thus, goat milk has a higher nutritional value than cow or sheep milk.

Milk fat is a critical component in the nutritional quality of dairy products. The molecular events associated with regulation of milk fat synthesis. For example, lipogenic genes including Acetyl-CoA carboxylase 1 (*ACACA)*, Fatty acid synthase (*FASN*), stearoyl-CoA desaturase (*SCD*), Fatty acid desaturase 1 (*FADS1*), *FADS2*, 1-acylglycerol-3-phosphate O-acyltransferase 6 (*AGPAT6*) and glycerol-3-phosphate acyltransferase, mitochondrial (*GPAM*) are increased until peak-lactation and decrease thereafter [4]. A deeper knowledge of lipid metabolism in the goat mammary gland during lactation is necessary to understand the features of milk, particularly the genes involved in fat metabolism.

MicroRNAs (miRNAs) are non-coding small RNAs that can post-transcriptionally regulate gene expression by pairing with the 3′-untranslated regions (3′-UTRs) or the coding regions of their target mRNAs. The base pairing between miRNA and target gene leads to either degradation of the mRNA or repression of protein translation [5]. Recently, miR-15a, miR-30e and miR-148a have been reported to regulate triacylglycerol synthesis in goat mammary epithelial cells (GMECs) by targeting low-density lipoprotein receptor-related protein 6 (*LRP6)*, yes-associated protein 1 (*YAP1*) and peroxisome proliferative activated receptor gamma coactivator 1 alpha (*PGC-1alpha*) [6, 7].

In the present study, we analyzed the miRNA expression patterns of 715 miRNAs using a highly sensitive method of S-poly(T) Plus miRNA real-time PCR [8, 9]. We found that miR-25 is implicated in lipid metabolism during lactation, by directly targeting peroxisome proliferative activated receptor gamma coactivator 1 beta (*PGC-1beta*), which modulates the expression of sterol regulatory element-binding proteins (*SREBPs*). Our results establish a miR-25/*PGC-1beta* regulatory axis in lipid metabolism during lactation.

## Methods

### Animal tissue samples

Three-year-old Xinong Saanen dairy goats from Northwest A&F University experimental farm were selected and sacrificed for mammary gland tissue collection. All selected goats were of similar body weight and in non-lactation, early lactation (15 d after parturition), peak lactation (60 d after parturition) or late lactation (120 d after parturition) periods. Mammary gland tissues were immediately snap-frozen in liquid nitrogen after washing in diethylpyrocarbonate (DEPC)-treated water. All experimental procedures involving dairy goats were approved by the Institutional Animal Care and Use Committee of the College of Animal Science and Technology, Northwest A&F University, Yangling, Shaanxi, China.

### Cell culture and transfection

Goat mammary epithelial cells (GMECs) were isolated from mammary gland tissue, and purified as previously reported [10]. These cells were treated in a lactogenic medium for 48 h to induce differentiation into secretary cells [11, 12]. For cell experiments, GMECs were cultured in DMEM/F12 medium (Hyclone Laboratories, Beijing, China), containing 5 μg/mL insulin, 5 μg/mL hydrocortisone, 100 U/mL penicillin, 100 μg/mL streptomycin, 10 ng/mL epidermal growth factor 1 (EGF-1, Gibco, Gaithersburg, MD, USA), and 10% fetal bovine serum (FBS, Biological Industries, BeitHaemek, Israel) in a humidified incubator with 5% CO_2_ at 37 °C. Synthetic miRNA mimics were purchased from RiboBio (Guangzhou, Guangdong, China) and transfected into GMECs using the K2 transfection system (Biontex Laboratories GmbH, München, Germany) according to manufacturer’s instructions.

### RNA extraction

Total RNA of tissues and cells was extracted with RNAiso Reagent (TaKaRa, Dalian, China) according to the manufacturer’s instructions. The quality of total RNA was checked by 1% agarose gel electrophoresis. The RNA was quantified using a NanoDrop 2000c Spectrophotometer (Thermo Fisher Scientific, Wilmington, DE, USA) and stored at − 80 °C before use.

### Real-time PCR

Mature miRNA expression level was determined using the S-Poly (T) plus method (Geneups, Shenzhen, Guangdong, China).

For miRNA, amplification conditions were as follows: a 10-μL reaction containing 0.2 μg total RNA, 2.5 μL 4× reaction buffer, 1 μL poly A/RT enzyme mix [with 0.8 units of Poly(A) polymerase and 100 units of M-MLV High Performance Reverse Transcriptase] and 1 μL 0.5 μmol/L RT primer. The reaction was performed at 37 °C for 30 min, followed by 42 °C for 30 min, then 75 °C for 5 min. The RT products were amplified and detected using a universal Taqman probe in a 20-μL PCR reaction containing 0.5 μL RT products, 4 μL 5× qPCR probe Mix, 0.5 units Hot Start Polymerase (FAPON, Shenzhen, Guangdong, China), 0.2 mmol/L universal Taqman probe, 0.5 μmol/L forward primer and 0.5 μmol/L universal reverse primer. Primers used were shown in Additional file 1: Table S1. 18S rRNA was used as an internal control. The primers used were as follows: 18S rRNA F: CAGCACATCTTGCGAGTACTC and 18S rRNA R: GTGCAGGGTCCGAGGTCAGAGCCACCTGGGCAATGCAGTGATGGCAAAGG.

For mRNA evaluation, 0.5 μg total RNA was synthesized into cDNA using M-MLV Reverse Transcriptase (TaKaRa) with oligo(dT) 18 plus random hexamer primers (Promega, Madison, Wisconsin, USA). Real-time PCR assays were performed with gene specific primers and SYBR Green PCR Master Mix (Applied Biosystems, Foster, CA, USA). The expression was normalized to ubiquitously expressed transcript ubiquitously expressed transcript protein (*UXT*).

The PCR reaction was performed at 95 °C for 3 min, followed by 40 amplification cycles consisting of 95 °C for 10 s and 60 °C for 30 s. All real-time PCRs were performed on an ABI StepOneplus real-time PCR System (Applied Biosystems). Primers used for real-time PCR are listed in Additional file 2: Table S2. Relative expression was calculated using the 2 ^-^^△△^^Ct^ method.

### Oil red O staining

Cells were washed three times with phosphate buffered saline (PBS) and then fixed in 10% paraformaldehyde for 1 h at 4 °C. After two washes with PBS, the cells were stained with Oil Red O (0.5 g Oil Red O in 100 mL 70% ethyl alcohol and filtered through a 0.2 μm filter) for 1 h. Cells were then washed thrice with PBS and photographed under a light microscope.

Subsequently, 400 μL of isopropyl alcohol was added to each well, and plates were oscillatedrapidly for about 5 min. Absorbance was then measured at 510 nm. The relative fat droplet content was normalized to the control, and the results of at least three independent experiments were combined.

### Triglyceride assay

The amount of intracellular triglyceride relative to total protein was detected using a tissue/cell triacylglycerol assay kit (Applygen Technologies, Beijing, China) and a BCA Protein Assay kit (Thermo Fisher Scientific, Wilmington, DE, USA), respectively.

### 3′-UTR luciferase reporter assay

We applied TargetScan (http://www.targetscan.org) to predict targets and miRNA binding sites. To generate reporter constructs for luciferase assays, the 3′-UTR of *PGC-1beta* was PCR amplified from goat genomic DNA and inserted into the pmirGLO dual-luciferase vector (Promega). The primers used were as follows: *PGC-1beta* WT-1, 5′-CCAGAATTCTCTTCTCCCCATTACACCTTGACCC-3′ (forward) and 5′-CCACTCGAGTCCATTTACCCTGTACCCCTGGACT-3′ (reverse); *PGC-1beta* WT-2,5′-CCAGAATTCGACTGTATCCACCAGCTACCCAGAT-3′ (forward) and 5′-CCACTCGAGATTCCTCAAGAAACAAAGTTGGGAG-3′ (reverse). To construct mutated 3′-UTR reporter vectors, the predicted miRNA binding sites were mutated by site-directed mutagenesis with the following primers: Muta,5′-CTTTATGTGGGAAGAGAACGTTATAGAAATCTGTCT-3′ (forward) and 5′-TCAGCGAGACAGATTTCTATAACGTTCTCTTCCCACA-3′ (reverse); Mut b,5′-ACCTGGCTAGTGCTTATGACGTTATTGTTTAAGCTGG-3′ (forward) and 5′-TGGGGCCCAGCTTAAACAATAACGTCATAAGCACTAG-3′ (reverse); Mut c,5′-GGAGGGGTTTACTGTAACACGTTATCTGGCAGCCCAG-3′ (forward) and 5′-CAGCAGCTGGGCTGCCAGATAACGTGTTACAGTAAAC-3′ (reverse). All constructs were confirmed by sequencing.

For *PGC-1beta* 3′-UTR luciferase assays, co-transfection of GMECs with *PGC-1beta* 3′-UTR or mutated *PGC-1beta* 3′-UTR reporter plasmids and miR-25 mimics or mimic control was performed with the PEI transfection reagent following the manufacturer’s instructions.

Cells were harvested 48 h after transfection and assayed for *Renilla* and firefly luciferase activity using the Dual Luciferase Reporter Assay System (Promega) with a luminometer Lumat3 LB9508 (Berthold Technologies, Bad Wildbad, Germany). Firefly luciferase activity was normalized to *Renilla* luciferase activity.

### Western blotting

For western blot analysis, cells were collected and lysed with ice-cold RIPA buffer (50 mmol/L Tris-HCl, pH 7.5; 150 mmol/L NaCl; 1% NP-40; 0.25% sodium deoxycholate, 1 mmol/L EDTA), supplemented with PMSF (Sigma-Aldrich, St. Louis, MO, USA).

Protein concentration was determined using a BCA Protein Assaykit (Thermo Scientific). Proteins were separated by SDS-PAGE, transferred to nitrocellulose membranes (Millipore, USA) and probed with the primary antibodies: polyclonal rabbit anti-PGC-1beta (Cat #22378-1-AP, 1:500 dilution; Proteintech Group) or polyclonal rabbit anti-GAPDH (Cat #HS-PP-0640, 1:1,000 dilution; Hanlin Biotech, Shijiazhuang, China). Polyclonal goat anti-rabbit IgG-HRP (Bio-Rad, Hercules, CA, USA) was used as secondary antibody. The protein bands were visualized using the chemiluminescent ECL western blot detection kit (Advansta, Menlo Park, CA, USA), and images were captured with the Tanon-5200 imaging system (Tanon, Shanghai, China).

### Statistical analysis

All results are expressed as the mean ± SD (standard deviation) of at least three triplicates for each treatment. Pairwise comparisons were performed with Student’s *t*-test using GraphPad Prism 5 software. A *P*-value of < 0.05 was considered statistically significant.

## Results

### Differential temporal expression of miRNAs during lactation

**Fig. 1:**
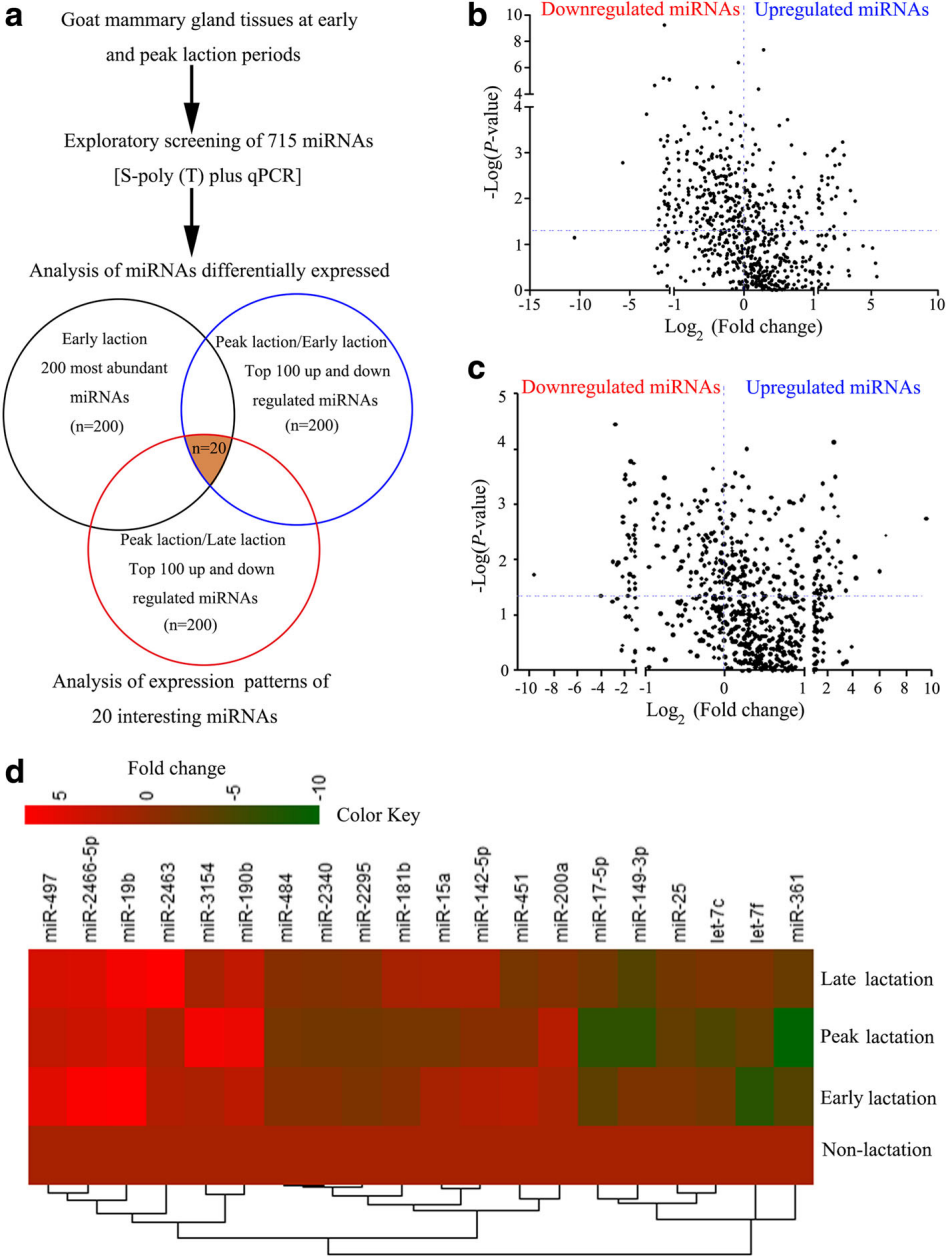
Quantitative real-time PCR analysis of differentially expressed miRNAs in goat mammary glands during lactation. 18S rRNA was used as an internal control. **a** Workflow of the identification of lactation-related miRNAs. **b**-**c** Volcano plot showing the differentially expressed miRNAs at peak lactation compared to early (**b**) or late (**c**) lactation. **d** Heatmap of the miRNAs that were found to be expressed differentially at the four lactation stages. Data for a single pool of three samples at non-lactation and early, peak and late lactation stages are shown. Red to green indicates high to low expression

**Fig. 2:**
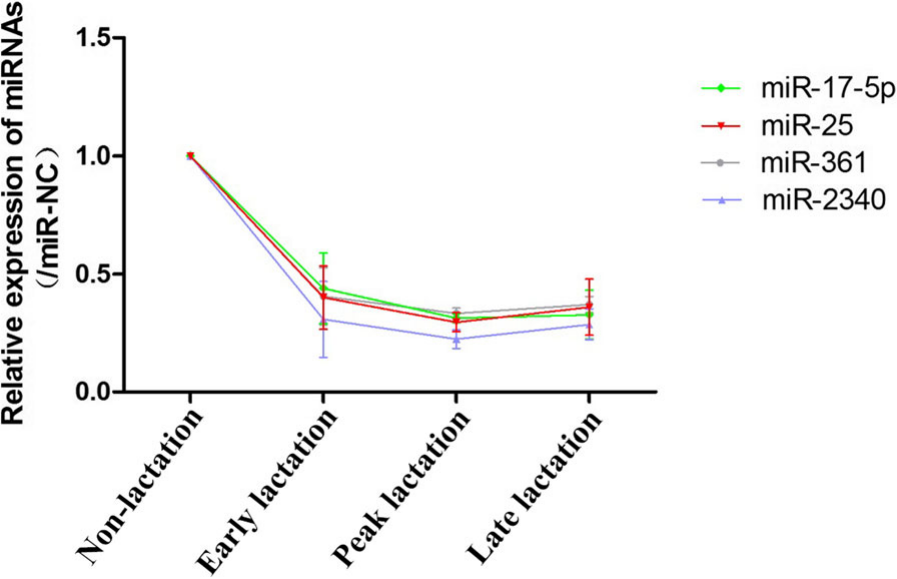
miR-25 expression is downregulated in goat mammary tissue from non-lactation to late lactation. Real-time PCR validated the expression levels of miR-17-5p, miR-25, miR-361 and miR-2340 in goat mammary tissue at non-lactation, early lactation, peak lactation and late lactation. The data were normalized to 18S. miR-25 expression at the non-lactation stage was set to 1.0. The error bars depict the mean ± S.D. of the samples with three goats at each stage

To explore potential miRNAs involved in the regulation of lactation, we analyzed the expression patterns of 715 miRNAs from goat mammary glands at four different stages, including non-lactation, early, peak and late lactation using the S-poly(T) plus miRNA quantitative real-time PCR method (Fig. 1a). Among these 715 miRNAs, 122, 143 and 450 are from the domestic goat, sheep and cow databases, respectively (Additional file 3: Table S3). We found that 107 miRNAs were differentially expressed between early and peak lactation (Fig. 1b), and 144 were differentially expressed between peak and late lactation (Fig. 1c). A total of 20 common miRNAs are in the 200 most abundant miRNAs and in the 100 most variable miRNAs across lactation stages (Fig. 1d). Of these 20 miRNAs, levels of miR-17-5p, miR-25, miR-361 and miR-2340 were decreased until peak-lactation and increased thereafter (Fig. 2). Chen et al. revealed that miR-17-5p regulated lipid metabolism during goat lactation [6]. In this study, we focused on miR-25, whose expression level was the highest among these four miRNAs (Additional file 4: Figure S1).

### miR-25 impaired triglyceride and lipid droplet accumulation in GMECs

**Fig. 3:**
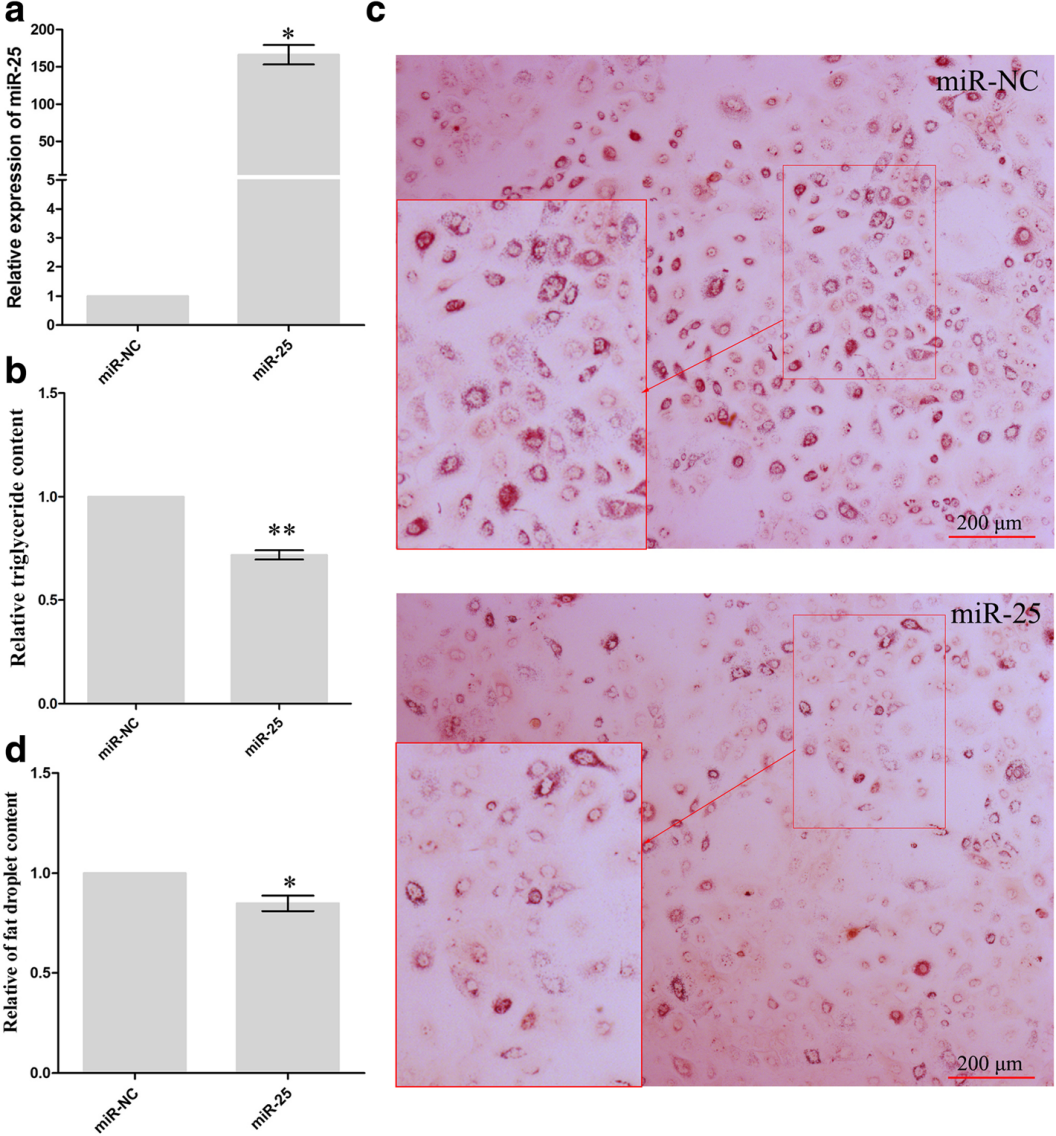
miR-25 represses intracellular triglyceride content and lipid droplet accumulation in GMECs. **a** Quantitative real-time PCR was performed to measure the expression level of miR-25 in GMECs 48 h after transfection with miR-25 mimics (miR-25), compared with mimic control (miR-NC). miR-17 was used as a positive control. The error bars depict the mean ± S.D. of at least three independent experiments. *P < 0.05. **b** Chemical analysis showing miR-25 overexpression leading to decreased triglyceride levels in GMECs. Data are shown as the mean ± S.D. of three independent cell samples. **P < 0.01. **c** Analysis of the presence of lipid droplets in GMECs treated for 48 h with or without miR-25 mimics followed by Oil Red O staining. Scale bar, 200 μm. **d** Oil Red O-based quantification of fat accumulation in GMECs. Each bar represents the mean ± S.D. for three samples. *P < 0.05

Triacylglycerol droplets and are stored in the cytoplasm as micro lipid droplets [13]. To access the function of miR-25 in lipid metabolism, we analyzed the regulation of triglyceride synthesis and of lipid drop accumulation by miR-25 in goat mammary epithelial cells. We transfected epithelial cells with miR-25 mimics or mimic control (miR-NC). The efficiency of miRNA mimic transfections was confirmed by real-time PCR (Fig. 3a). We found that, miR-25 significantly reduced (0.72-fold, *P* = 0.0064) the synthesis of triglyceride relative to control (Fig. 3b). Moreover, Oil Red O staining assays showed a reduced number of lipid droplets in miR-25 transfected cells compared to the control (Fig. 3c). Further quantification of lipid droplets confirmed that miR-25 significantly reduced lipid droplet accumulation (0.85-fold, *P* = 0.0213, Fig. 3d). Taken together, our results show that miR-25 has a repressive role in milk lipid metabolism.

### miR-25 repressed the expression of PGC-1beta

**Fig. 4:**
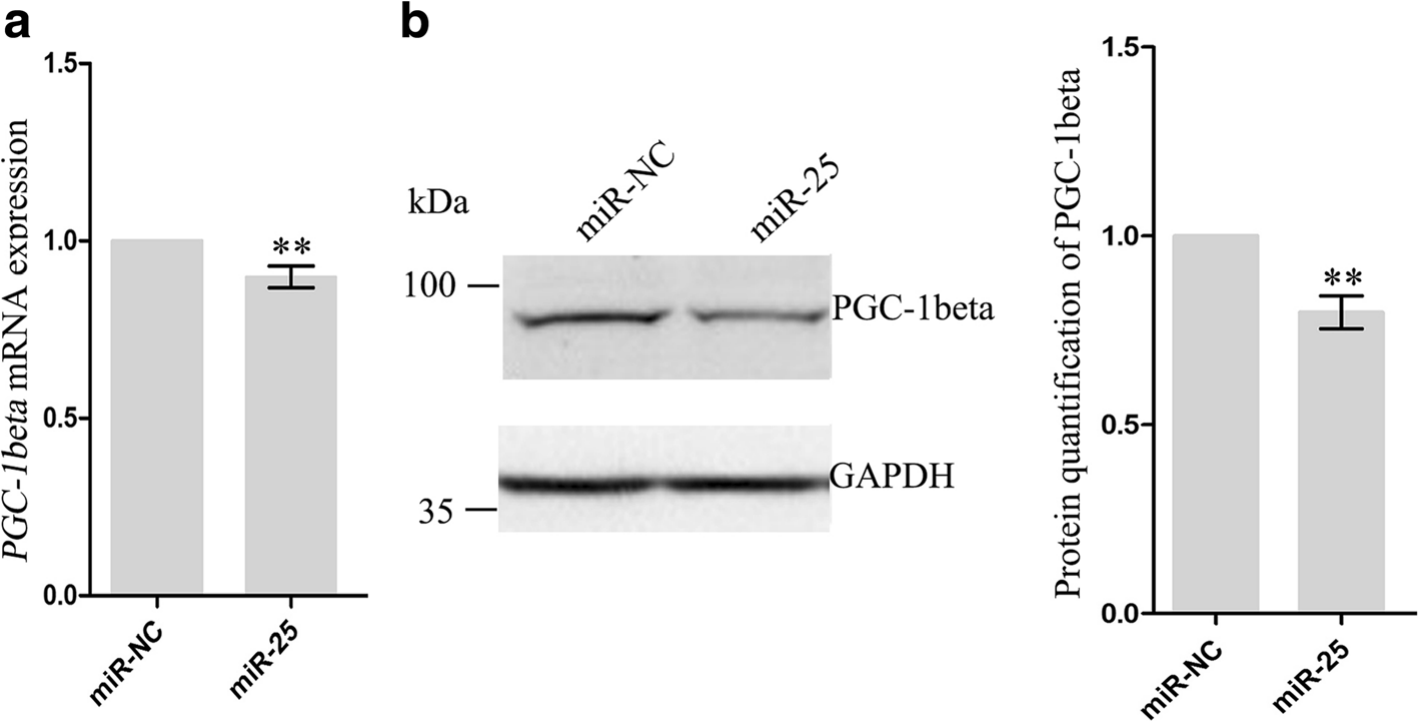
*PGC-1beta* transcript and protein levels in GMECs transfected with miR-25 mimics or mimic control (miR-NC). **a** Quantitative real-time PCR was performed to analyze the mRNA levels of *PGC-1beta* in GMECs using *UTX* as an internal control. (**b**, left) Western blot for PGC-1beta in GMECs. (Right) Quantification of PGC-1beta protein levels. miR-25 reduced PGC-1beta protein levels in GMECs. Data represent the mean ± SD of three independent cell samples. **P < 0.01

We next explored the mechanism through which miR-25 regulates lipid metabolism by searching for mRNA targets that might mediate its effects in mammary epithelial cells. TargetScan 7.1 (Cambridge, MA, USA) predicted 1,038 genes as its target genes. Further Gene Ontology enrichment analysis using DAVID 6.8 (https://david.ncifcrf.gov) showed that 30 of these genes are lipid metabolism-associated genes (Additional file 5: Table S4). Peroxisome proliferative activated receptor gamma coactivator 1 beta (*PGC-1beta*) is a well-known regulator of lipogenic genes and is required for the full transcriptional activity of SREBP [13]. To investigate whether *PGC-1beta* is a potential target gene of miR-25, 48 h after transfection of cells with miR-25 mimic or its control, we analyzed the mRNA and protein levels of PGC-1beta. Due to gradual increases in RNA concentration during lactation, we used *UXT* as a reference, which is expressed stably across time [14]. We found that miR-25 overexpression resulted in a reduction in the level of *PGC-1beta* mRNA (0.90-fold, *P* = 0.0284, Fig. 4a), while western blotting revealed post-transcriptional suppression of PGC-1beta by miR-25 (0.8-fold, *P* = 0.0155, Fig. 4b). These data demonstrate that *PGC-1beta* is a probable downstream target of miR-25.

### miR-25 regulates the PGC-1beta gene by directly targeting its 3′-UTR

**Fig. 5:**
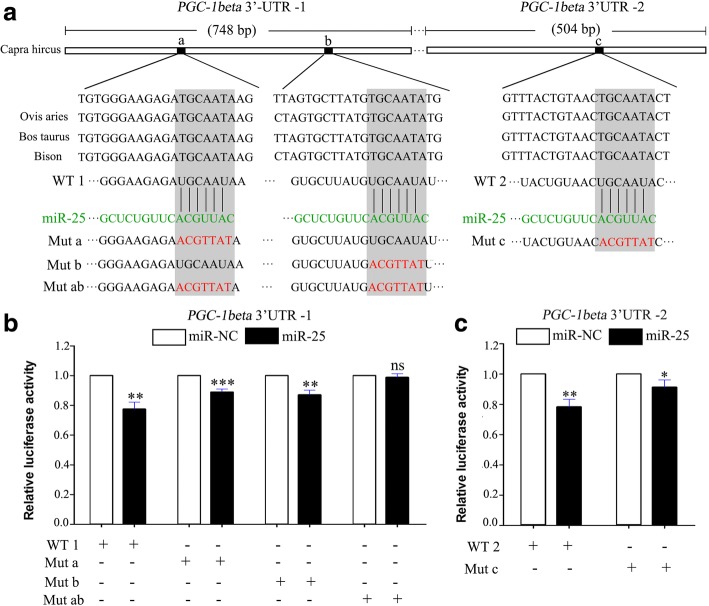
miR-25 downregulated *PGC-1beta* protein expression by directly targeting its 3′-UTR. (**a**) Schematic illustration of the predicted binding sites of miR-25 in the 3′-UTR of *PGC-1beta*. Sequence conservation of three predicted miR-25 binding sites within the 3′-UTR of *PGC-1beta* in four ruminates (*Capra hircus, Ovisaries, Bostaurus* and bison). WT1 and WT2 represent, respectively, *PGC-1beta* 3′-UTR-1 (748bp) and *PGC-1beta * 3′-UTR-2(504bp). The wild type (WT) miRNA-binding sites (black) were modified to complementary sequences (red) to construct the mutated *PGC-1beta* 3′-UTR (Mut). Mut a, b, c and ab corresponds to mutated *PGC-1beta* 3′-UTR in site 1, 2, 3 and double mutation of sites 1 and 2, respectively. (**b**-**c**) Luciferase reporter assay to assess interaction between miR-25 and *PGC-1beta* 3′-UTR-1 (**b**) and *PGC-1beta* 3′-UTR-2 (**c**) in GMECs. Data represent the mean ± S.D. of three independent cell samples. *P < 0.05, **P < 0.01, ***P < 0.001

To understand how miR-25 affects the expression of *PGC-1beta*, we determined whether there is a direct interaction between miR-25 and *PGC-1beta*. There are three predicted miR-25 binding sites at the 3′-UTR of *PGC-1beta* mRNA (sites a, b and c), which are well conserved between goat, sheep, cattle and bison (Fig. 5a). As shown in Fig. 5a, we constructed two wild-type *PGC-1beta* 3′-UTR luciferase reporter plasmids (WT 1 and 2), and generated their corresponding mutant constructs (Mut a, b, ab and c) with seven mutated residues in the predicted binding sites by site-directed mutagenesis. Enhanced expression of miR-25 significantly repressed the luciferase activities of WT1 and WT2 reporters (0.77-fold, *P* = 0.0012 and 0.78-fold, *P* = 0.0017, respectively), whereas these repressions were partly abrogated by Mut a, b or c (Fig. 5b). Moreover, the repression of WT1 was completely abolished when both site a and b were mutated (Fig. 5c). Based on these results, we conclude that miR-25 repressed *PGC-1beta* expression in goat mammary epithelial cells by directly targeting its 3′-UTR.

### miR-25 modulated other lipid metabolism-related genes in GMECs

**Fig. 6:**
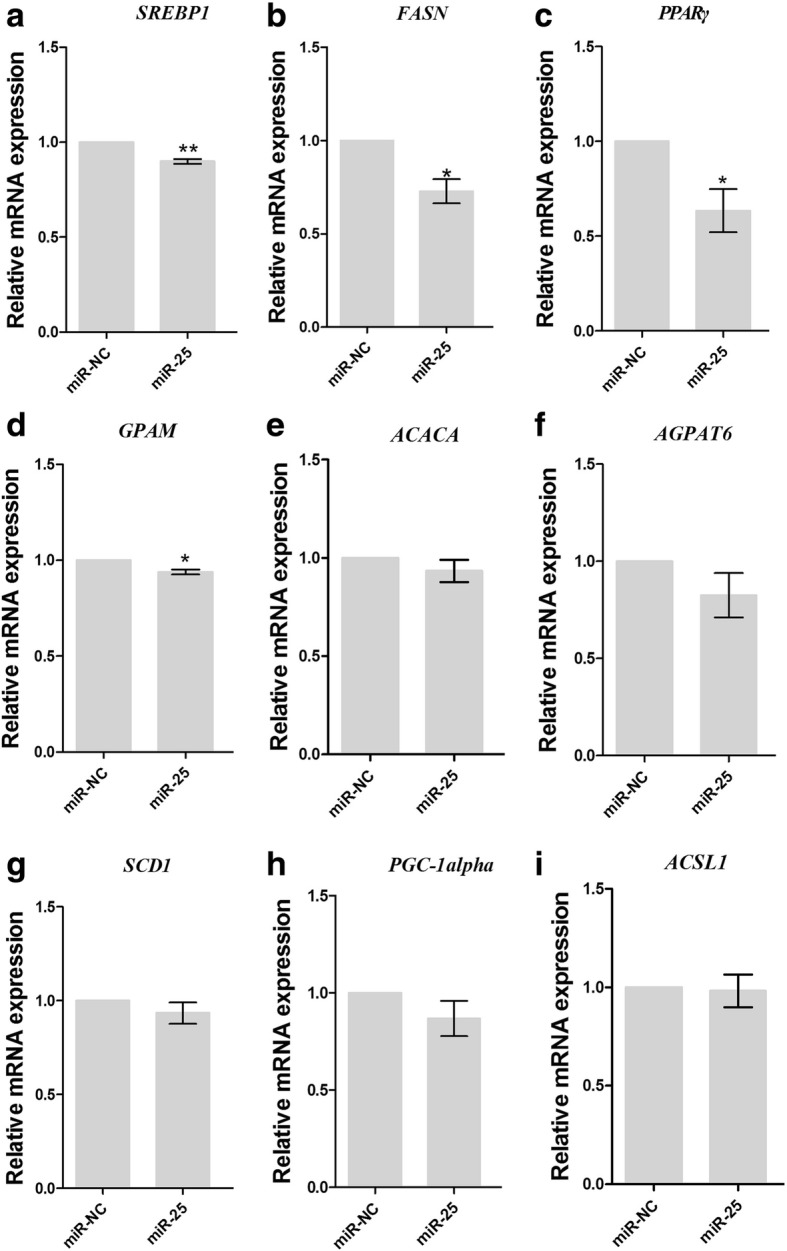
Regulation of *SREBP1*, *FASN*, *PPARG*, *GPAM*, *ACACA*, *AGPAT6*, *SCD1*, *PGC-1A* and *ACSL* transcript levels in GMECs transfected with miR-25 mimics or mimic control (miR-NC). Cells were collected 48 h after transfection and RNA was isolated and analyzed for mRNA levels of *SREBP1* (**a**), *FASN* (**b**), *PPARG* (**c**), *GPAM* (**d**), *ACACA* (**e**), *AGPAT6* (**f**), *SCD1* (**g**), *PGC-1A* (**h**) and *ACSL* (**i**) by quantitative real-time PCR. For data normalization, *UXT* was used as an endogenous control. Transcript expression levels are shown as fold change. The data represent the mean ± S.D. of three independent cell samples. * *P* < 0.05, ** *P* < 0.01

Bionaz and Loor [4] identified a regulatory network in lipid metabolism with sterol regulatory element-binding protein 1 (*SREBP1*), sterol regulatory element-binding protein 2 (*SREBP2)* and peroxisome proliferator-activated receptor gamma (*PPARG*) as core regulatory genes. In the *SREBP1* subnetwork, PGC-1beta co-activates the *SREBP* transcription factor and induces the expression of lipogenic genes [13]. Here, we measured the impact of miR-25 overexpression on nine genes in the *SREBP1* subnetwork (Fig. 6). miR-25 overexpression had no significant effect on *SCD1*, *ACACA*, *AGPAT6*, insulin induced gene 1 (*INSIG1*) or *PGC-1alpha*. However, we found that miR-25 reduced the mRNA levels of *SREBP1* (0.86-fold, *P* = 0.0031), *FASN* (0.83-fold, *P* = 0.0368), *GPAM* (0.92-fold, *P* = 0.0014) and *PPARG* (0.75-fold, *P* = 0.0325).

## Discussion

In this study, we analyzed the expression of 715 miRNAs in the goat mammary gland during lactation and characterized miR-25 as a repressor in lipid-metabolism. Our results revealed that miR-25 overexpression leads to repression of *PGC-1beta* by direct targeting of three different regions within the *PGC1-beta* 3′-UTR. This reduces the expression of certain lipogenic genes, triglyceride synthesis and lipid droplet accumulation.

**Fig. 7:**
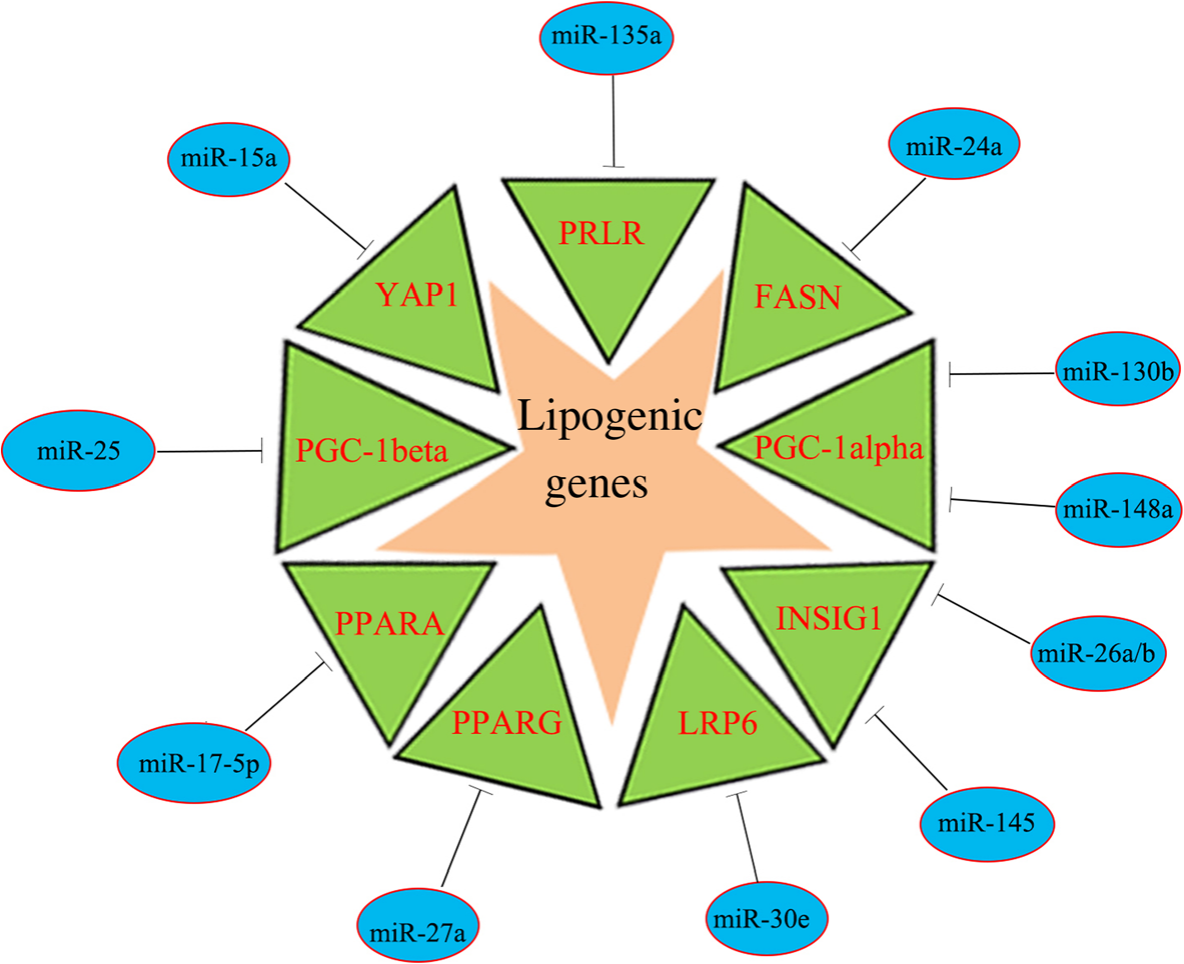
Experimentally identified lipid metabolism-related miRNAs that target lipogenic genes during lactation in goat

During the process of lactation, triacylglycerol accumulation and fatty acid synthesis are essential events that occur in every stage. Twelve miRNAs (miR-15a, miR-17-5p [15], miR-24 [16], miR-26a/b [17], miR-27a [18], miR-30e-5p, miR-130b [19], miR-135a [20], miR-145 [21] and miR-148a [15]) have been shown to repress genes involved in lipid metabolism, including prolactin receptor (*PRLR*), *FASN*, *LRP6*, *YAP1*, *PPARG*, *INSIG1* and *PGC-1alpha* during lactation in goat. Evidence suggests that one miRNA may regulate multiple genes, while one gene may be targeted by multiple miRNAs; for example, miR-17 targets *PPARA* [6], while miR-130b, miR-26a/b and miR-154 target *INSIG1*. miR-25 joins this miRNA regulatory network as a repressor of lipid synthesis (Fig. 7). Undoubtedly, the functional roles of other unstudied miRNAs remain to be explored.

miR-25 belongs to the miR-25-93-106b cluster, which plays an important role in many malignancies, including ovarian cancer [22], cervicalcancer [23], cholangiocarcinoma [24] and lung cancer [25, 26]. There is growing evidence that miR-25 is involved in lipid metabolism. Liang et al. [27] showed that miR-25 is downregulated during adipocyte differentiation and suppressed 3 T3-L1 adipogenesis by targeting Kruppel-like factor 4 and CCAAT/enhancer-binding protein alpha. Moreover, Hsieh et al. found that miR-25 was downregulated in high-fat diet fed mice relative to low-fat diet fed mice [15]. When viewed together, these findings and our results provide strong evidence to support the involvement of miR-25 in lipid metabolism.

*PGC-1beta* has long been shown to stimulate the expression of genes involved in lipid metabolism via direct co-activation of the *SREBP* family [13]. In the present study, decreased *SREBP-1c* expression by miR-25 overexpression suggests that miR-25 modulates lipid metabolism, at least partially, via the PGC-1beta/SREBP pathway. On the other hand, miR-25 represses target *PGC-1beta* by binding to multiple sites within its 3′-UTR. Multiple binding sites for the same miRNA can enhance the degree of modulation [14]. In addition, the three miR-25 binding sites within the *PGC-1beta* 3′-UTR are highly conserved among ruminants, highlighting miR-25 as regulator of PGC-1beta protein expression. These data indicate the importance of miR-25 in regulating *PGC-1beta* expression.

## Conclusion

In conclusion, we revealed miRNA expression patterns in goat mammary gland tissue during lactation and identified miR-25 as lactation related miRNA. We then characterized the role of miR-25 in triglyceride and lipid droplet accumulation and lipid metabolism-related gene expression in GMECs, and determined that miR-25 can repress lipid synthesis via *PGC-1beta* in GMECs during lactation.

## Additional files

Additional file 1: **Table S1.** Primers used for miRNA real-time PCR. (XLSX 58 kb)

Additional file 2: **Table S2.** Primers used for real-time PCR. (DOCX 18 kb)

Additional file 3: **Table S3.** List of 715 studied miRNAs. (XLSX 26 kb)

Additional file 4: **Figure S1.** Expression levels of miR-17-5p, miR-25, miR-361 and miR-2340 in goat mammary tissue at non-lactation, early lactation, peak lactation and late lactation. Data was presented as Ct value. (DOCX 65 kb)

Additional file 5: **Table S4.** 30 lipid metabolism-associated genes. (XLSX 13 kb)

## Abbreviations

ACACA: Acetyl-CoA carboxylase 1
AGPAT6: 1-acylglycerol-3-phosphate O-acyltransferase 6
FADS1: Fatty acid desaturase 1
FASN: Fatty acid synthase
GMEC: Goat mammary epithelial cells
GPAM: Glycerol-3-phosphate acyltransferase, mitochondrial
INSIG1: Insulin induced gene 1
LRP6: Low-density lipoprotein receptor-related protein 6
PGC-1beta: Peroxisome proliferative activated receptor gamma coactivator 1 beta
PPARG: Peroxisome proliferator-activated receptor gamma
PRLR: Prolactin receptor
SCD: Stearoyl-CoA desaturase
SREBP1: Sterol regulatory element-binding protein 1
SREBP2: Sterol regulatory element-binding protein 2
SREBPs: Sterol regulatory element-binding proteins
UXT: Ubiquitously expressed transcript protein
YAP1: Yes-associated protein 1
