# Supplementary material for: miR-25 modulates triacylglycerol and lipid accumulation in goat mammary epithelial cells by repressing PGC-1beta
Source: J Anim Sci Biotechnol. 2018 Jun 18;9:48. doi: 10.1186/s40104-018-0262-0 (PMC6004671; doi:10.1186/s40104-018-0262-0)
Supplement: Supplementary file 2 — Table S2. Primers used for real-time PCR. (DOCX 18 kb) [file 40104_2018_262_MOESM2_ESM.docx]

Table S2. Primers used for real-time PCR.

| **Gene** |  | **Forward Primer (5’-3’)** | **Reverse Primer (5’-3’)** |
| --- | --- | --- | --- |
| *PPARγ* | *HQ589347.1* | CCTTCACCACCGTTGACTTCT | GATACAGGCTCCACTTTGATTGC |
| *FASN* | *DQ915966.3* | GGGCTCCACCACCGTGTTCCA | GCTCTGCTGGGCCTGCAGCTG |
| *ACACA* | *XM_005693156.1* | CTCCAACCTCAACCACTACGG | GGGGAATCACAGAAGCAGCC |
| *GPAM* | *AY515690* | GCAGGTTTATCCAGTATGGCATT | GGACTGATATCTTCCTGATCATCTT |
| *PGC1α* | *XM_018049155.1* | GTACCAGCACGAAAGGCTCAA | ATCACACGGCGCTCTTCAA |
| *AGPAT6* | *NM_001083669.1* | AAGCAAGTTGCCCATCCTCA | AAACTGTGGCTCCAATTTCGA |
| *SCD1* | *GU947654* | CCATCGCCTGTGGAGTCAC | GTCGGATAAATCTAGCGTAGCA |
| *ACSL1* | *BC119914* | TGACTGTTGCTGGAGACTGG | CAGCCGTCTTTATCCAGAGC |
| *SREBP-1* | *HM443643.1* | ACGCCATCGAGAAACGCTAC | GTGCGCAGACTCAGGTTCTC |
| *PGC1b* | *XM_018050171.1* | TCCTGCAAGAGCCCGGAGTAT | GGCTCTGGTAGGGGCAGTG |
| *UXT* | *XM_005700842.1* | CAGCTGGCCAAATACCTTCAA | GTGTCTGGGACCACTGTGTCAA |
