# Supplementary material for: miR-25 modulates triacylglycerol and lipid accumulation in goat mammary epithelial cells by repressing PGC-1beta
Source: J Anim Sci Biotechnol. 2018 Jun 18;9:48. doi: 10.1186/s40104-018-0262-0 (PMC6004671; doi:10.1186/s40104-018-0262-0)
Supplement: Supplementary file 4 — Figure S1. Expression levels of miR-17-5p, miR-25, miR-361 and miR-2340 in goat mammary tissue at non-lactation, early lactation, peak lactation and late lactation. Data was presented as Ct value. (DOCX 65 kb) [file 40104_2018_262_MOESM4_ESM.docx]

Figure S1


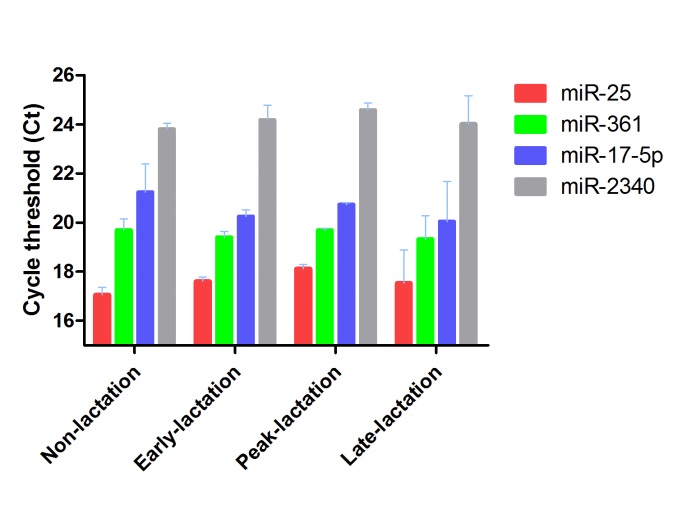


Figure S1. Expression levels of miR-17-5p, miR-25, miR-361 and miR-2340 in goat mammary tissue at non-lactation, early lactation, peak lactation and late lactation. Data was presented as Ct value.
